# COVID-19 Vaccine Challenges in Developing and Developed Countries

**DOI:** 10.7759/cureus.23951

**Published:** 2022-04-08

**Authors:** Harshani Yarlagadda, Meet A Patel, Vasu Gupta, Toram Bansal, Shubekshya Upadhyay, Nour Shaheen, Rohit Jain

**Affiliations:** 1 Internal Medicine, Avalon University School of Medicine, Willemstad, CUW; 2 Internal Medicine, Tianjin Medical University, Tianjin, CHN; 3 Internal Medicine, Dayanand Medical College and Hospital, Ludhiana, IND; 4 Internal Medicine, University of Mumbai, Mumbai, IND; 5 Internal Medicine, University of California Berkeley, Berkeley, USA; 6 Internal Medicine, Alexandria University, Alexandria, EGY; 7 Internal Medicine, Penn State Health Milton S. Hershey Medical Center, Hershey, USA

**Keywords:** covid-19, vaccine affordability, vaccine acceptability, herd immunity, pandemic, vaccine

## Abstract

The coronavirus disease 2019 (COVID-19) pandemic has led to significant psychological and economical distress. Within a year after COVID-19 was declared a pandemic, several vaccines against COVID-19 were approved for emergency use. The journey from vaccine discovery to global herd immunity against COVID-19 continues to present significant challenges revolving around its development, affordability, accessibility, and acceptability at both a country level and an individual level. The main challenge faced by developed countries is the acceptability of the COVID-19 vaccine and the main challenge faced by developing countries is the affordability and accessibility of the COVID-19 vaccine.

## Introduction and background

Coronavirus disease 2019 (COVID-19) was declared a global pandemic on March 11, 2020, by the World Health Organization (WHO), and less than a year later, there were many new announcements regarding vaccines. There was a great deal of importance surrounding the discovery, development, and distribution of vaccines worldwide. Approximately 200 vaccine candidates underwent preclinical trials and evaluations in December 2020 [1]. There were various vaccine candidates from different countries across the globe who were granted authorization for emergency use [2]. On October 6, 2020, the FDA and the WHO approved the emergency use authorization to allow for rapid vaccine roll-out in countries around the globe [3].

The spread of the COVID-19 pandemic caused the world to experience significant psychological and economic distress. The virus transmission can be limited by using face coverings, physical distancing, contact tracing, and social restrictions. However, these methods are insufficient to end the COVID-19 pandemic [4]. The most effective approach is administering vaccines to an enormous number of the world's population to achieve herd immunity [4]. Hence, herd immunity has to attain a high enough threshold immunity to protect most people in a particular geographic area for a specific period [5]. Vaccination has been shown to decrease hospitalization, the severity of illness, and death from COVID-19 [6]. Several challenges arise when this type of immunity is required since numerous social and economic factors can cause hindrances. Table 1 provides an overview of the top 15 highest vaccinated countries. While Table 2 shows an overview of the 15 lowest vaccinated countries. All the data in the table were last accessed on February 25, 2022.

**Table 1:** The top 15 vaccinated countries * According to The New York Times, the data are compiled from the University of Oxford’s “Our World in Data” project [7].

Top 15 highest vaccinated countries*
United Arab Emirates (96%)
Brunei (93%)
Malta (91%)
Portugal (90%)
Chile (90%)
China (88%)
Cuba (87%)
South Korea (85%)
Singapore (85%)
Cambodia (84%)
Spain (82%)
Denmark (81%)
Canada (81%)
Malaysia (81%)
Australia (80%)

**Table 2:** The bottom 15 vaccinated countries * According to The New York Times, the data are compiled from the University of Oxford’s “Our World in Data” project [7].

15 lowest vaccinated countries*
Burundi (0.1%)
Congo (0.2%)
Haiti (0.8%)
Chad (0.9%)
Yemen (1.2%)
Ethiopia (1.4%)
South Sudan (2.5%)
Cameroon (2.6%)
Papua New Guinea (2.7%)
Nigeria (2.7%)
Tanzania (3%)
Mali (3.2%)
Madagascar (3.4%)
Burkina Faso (3.8%)
Malawi (4.3%)

The challenges that arise with COVID-19 vaccine uptake include the issues of distribution, affordability, accessibility, and acceptability at both a country level and an individual level.

## Review

A thorough review of the literature was done using a PubMed search. Different keywords like COVID-19 vaccine, challenges of vaccination, development, distribution, affordability, accessibility, and acceptability of vaccines were used to write this review.

Development

As widespread immunity requires the development and distribution of vaccines, this includes ensuring that safe and effective vaccines are produced continuously and delivered to all countries [2]. The massive scale of COVID-19 vaccine doses currently needed has never been seen in the past. To fully vaccinate 70% of the world population, approximately 11 billion COVID-19 doses would be required [8]. The production turnaround had to be at its maximum capacity to meet the worldwide demand, which placed significant pressure on the global supply chains as they were completely blindsided by the pandemic [4].

Developing the COVID-19 vaccine in a very short amount of time was also very challenging, as it needed to be safe and effective but also affordable. In the US, the Operation Warp Speed program was developed to accelerate the development of vaccines. High-income countries poured billions of dollars into the production and acquisition of vaccines, which meant unfair allocation of vaccines to low-income countries (LICs) [9]. In September 2021, the WHO set a target of vaccinating 70% of the world population but less than 1% of the population of lower-income countries and 10% in lower-middle-income countries have been vaccinated against more than 50% of the population belonging to high-income countries [10]. According to a report released by the United Nations (UN) on March 28, 2022, although the distribution of vaccines has risen worldwide, so has inequality. Of the 10 billion doses distributed worldwide, only 1% went to lower-income countries, which meant that almost 2.8 billion people are still waiting for their first dose [11]. Even the COVID-19 Vaccines Global Access (COVAX) failed to procure and allocate vaccines for lower-income countries [12]. To prevent this to a certain extent, AstraZeneca and Serum Institute of India collaborated to produce and supply vaccines for low and middle-income countries. Various funds, for example, PM Cares Fund in India [13], and task forces, such as the African Vaccine Acquisition Task Team of the African Union in Africa, were set up to develop and acquire vaccines [14].

Immediate access to vaccines and funding can help the poorest of the countries to fight this pandemic with various international organizations like Global Dashboard for Vaccine Equity and the WHO helping these countries to develop the right strategies that are best suited to their citizens.

Affordability

COVAX was an international initiative founded in April 2020; it aims to assure equitable access to the COVID-19 vaccines around the globe. Low-income and middle-income countries (LMICs) heavily depend on COVAX for availability; therefore, COVAX intends to distribute to these countries in quantities enough to vaccinate at least 20% of the populations in LICs of Asia, Africa, and Latin America by the end of 2021 [15]. The initiative acquired about 700 million doses, which is only enough to vaccinate about 10% of the population in 67 LICs [16]. According to the World Bank Group, a low-income country has an economy of $1005 or less gross national income (GNI) per capita, whereas a middle-income country has an economy of $1006 to $3955 GNI per capita. These LMICs make up about 85% of the world's population [4].

LMICs were able to purchase the vaccines at an average of $1.60-$2.00 per dose, which was a markedly lower price in contrast to high-income countries that were paying around $11 per dose [4]. Even though COVAX intended to provide equitable access to all the countries, high-income countries, which make up about 16% of the world's population, made pre-order deals with vaccine developers. These high-income countries pre-ordered about 4.2 billion COVID-19 vaccine doses, equivalent to about 70% of the available quantity in 2021 [4]. This led to around 67 LICs being at the unfair disadvantage of not being able to vaccinate at least 90% of their populations [16]. These LMICs require support from high-income countries to achieve equitable access to vaccines. The G7 leaders pledged to provide extra doses to LIMCs by 2022 [8]. The United States and the United Kingdom agreed to donate 500 million and 100 million doses each. France, Germany, and Japan followed by pledging around 30 million doses each [8].

The pandemic has disproportionately affected the LIMCs and continues to remain a huge threat unless a larger population of the world is vaccinated. The costs of vaccine manufacture and delivery to these countries are enormous and represent a major barrier to reducing future COVID-19 waves through continued viral mutations. According to the United Nations Children's Fund (UNICEF), COVAX Readiness and Delivery Working Group on Delivery Costs estimated that it would cost approximately US$ 2 billion to deliver two doses of COVID-19 vaccine to 20% of the population in the 92 Advance Market Commitment (AMC) countries. A sub-group analysis showed that the Covishield vaccine manufactured by the Serum Institute of India would cost $2.88 per dose for India and $4.00 for Bangladesh. Covaxin produced by Bharat Biotech would cost Nepal's private market a staggering $35.00 per dose whereas Covishield would cost $4.00 per dose. The costs include, for example, capital cost adjustments, technical assistance, cold storage, human resources, and delivery costs among others [17].

Accessibility

Some areas posed some difficulty for vaccine access. The geography of many LICs presents a substantial hurdle to vaccine access. Regions like Nepal, Afghanistan, Pakistan, and Bhutan are located at high altitudes, making it extremely difficult to set up vaccination camps for community immunization [15]. An estimated 160 million people in Yemen, Syria, Ethiopia, and South Sudan are at liability for vaccine inaccessibility due to being located in isolated areas experiencing war, instability, and conflict [15].

**Figure 1:**
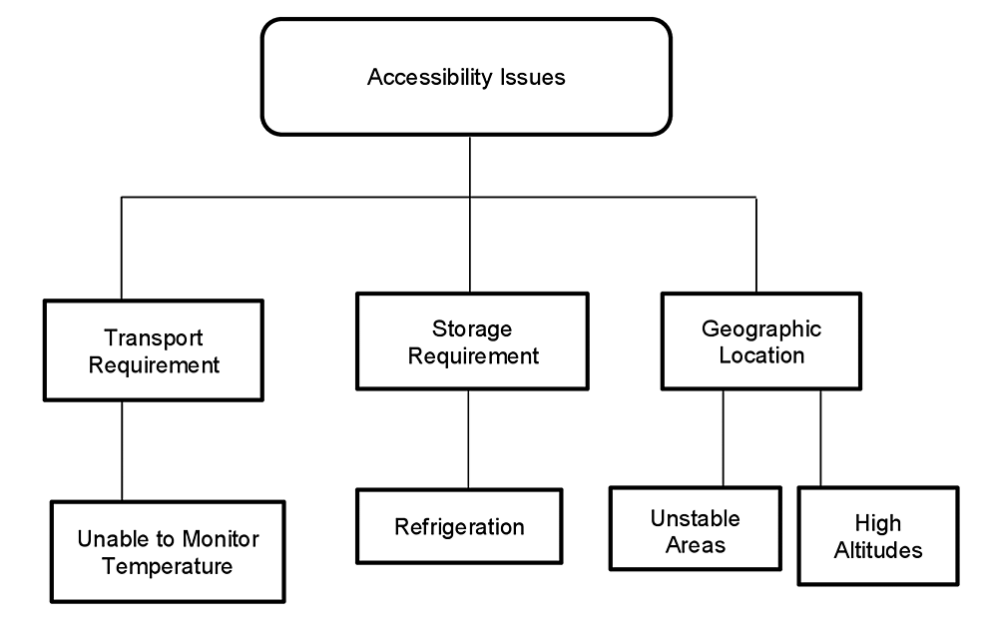
Specific accessibility issues causing difficulty in obtaining the vaccine

The transportation and storage requirements of the COVID-19 vaccine also cause a challenge in distribution. The Pfizer vaccine earlier required a refrigeration temperature of −60°C to −80°C, and the Oxford-AstraZeneca vaccine requires 2-8°C for storage and transportation [15]. This posed a huge challenge to distribute these vaccines to rural areas where infrastructure to maintain these temperatures are not available [18]. To ease the distribution process, on February 25, 2021, the US Food and Drug Administration (US FDA) announced the transportation and storage of vaccines at a more flexible temperature, which can be easily maintained in a pharmaceutical freezer [19]. Many LICs cannot still maintain and monitor these temperatures during transport and storage due to insufficient infrastructure [20]. An overview of the accessibility issues is illustrated in Figure 1.

Acceptability

The newly synthesized vaccine raises concern regarding acceptability among the public. Surveys were conducted to understand the consensus of the public and develop strategies for immunization programs. A 19-country global survey deduced that 71.5% of participants were acceptable to the COVID-19 vaccine [1]. Although there were individuals who were ready to accept the vaccine, many others were hesitant as they were afraid of the side effects [1]. In another survey, it was observed that respondents with higher income were more accepting of the COVID-19 vaccine than those with lower income [21]. A nationwide survey done in China reported that although most of the respondents were accepting of the vaccine, they were more inclined toward domestically produced vaccines rather than imported ones [22].

The hesitancy regarding the vaccine was more aggravated when misconceptions in social media and the internet led to people’s perceptions being negatively impacted [1]. A study examining the opinions of 100 million Facebook users found that the anti-vaccine group of people was fewer in number compared to the pro-vaccine group. However, the anti-vaccine group was more dominant on social media platforms and was more vocal and interactive with the undecided group [23]. Amongst the popular and widely spread COVID-19 vaccine conspiracy theory was that vaccines contained a microchip that the government would use to track individuals [23]. In a survey done in Nigeria, observers listed 33 different conspiracy theories regarding the COVID-19 virus and the vaccine [24]. Some believed COVID-19 was a biological weapon whereas others heard that vaccination was a way for the government to take their money [24]. Other social media-cultivated misinformation was that the COVID-19 mRNA vaccine causes infertility, which was later denied in a joint statement by the leading medical societies of the US including the American College of Obstetricians and Gynecologists [25]. This obscure, false rumor was believed by many, which led to further fear and reluctance to take the vaccine.

A 45,000-participant survey conducted between June 2020 and January 2021 in 12 countries found that people in LMICs were less hesitant to receive the vaccine than in countries like the United States and Russia [26]. A total of 80% of the surveyed individuals in Asia, Africa, and South America were ready to take the vaccine, whereas only 30% in Russia and 65% in the United States were equally ready [27].

Vaccine hesitancy has been an issue even before the pandemic but now has been a more significant concern. The 5C model of factors of vaccine hesitancy further explains some reasons for reluctance. These factors include confidence, complacency, constraints, calculation, and collective responsibility [28]. This 5C model evaluates the psychological antecedents of vaccination, which determine whether or not the person accepts vaccination. A cross-sectional study involving three Arabic countries concluded that by studying these five psychological antecedents of vaccines, an insight into vaccine hesitancy can be gained [29].

The first “C” is confidence, which refers to the trust in the efficacy and safety of the vaccine [30]. This trust also carries through to the healthcare system and the healthcare providers. Decreased confidence is related to apprehension about the medical benefits and doubt about the healthcare services. A cross-sectional study that includes responses from over 3400 healthcare workers showed that the acceptability of the vaccine increased with age, income level, and education [31]. The top three concerns among these healthcare workers were the speed of development/approval, the safety, and the effectiveness of the vaccine; about 70% of the participants were concerned about these factors [31]. The speed with which the vaccines were developed and brought into the market was unheard of, raising many doubts among the public, leading them to believe that the clinical trials were rushed, not holding the regulatory requirements to the highest standard [4].

The second “C”, complacency, is when an individual has a greater interest in immediate outcomes and has more risk-seeking behavior. In LICs where vaccine-preventable infections are still causing widespread deaths, their acceptance of vaccines is more than in high-risk countries that have already eradicated or eliminated many of these diseases. Hence, causing complacency, altered risk calculations, and decreased collective responsibility [28].

Constraints refer to the barriers that negatively impact the intention to receive the vaccine. These barriers could be psychological or structural, such as limited access to healthcare or lack of self-control, causing one to feel overwhelmed with daily life to even consider vaccination [30].

The calculation indicates an individuals’ analytical comparison between the risk of the infection and the risk of the vaccination to make a knowledgeable decision [30]. Individuals with a higher risk calculation tend to recognize higher risks linked to receiving the vaccination. Collective responsibility refers to contributing to herd immunity by being willing to vaccinate themselves to protect other members of their community [30]. A combination of these five factors could provide insight into an individual’s hesitancy toward the vaccine.

## Conclusions

The COVID-19 pandemic has led to a global health crisis and completely changed the lives of individuals all around the world. To cope with the pandemic, many vaccines for COVID-19 have been developed over the past two years because vaccination of the world's population is the most effective approach to achieving herd immunity. However, when this type of immunity is required, various social and economic factors can cause hindrances. According to the current literature, the challenges that arise with the COVID-19 vaccine include the following: (i) the development and production of sufficient doses required to vaccinate the entirety of the world's population; (ii) making the vaccines affordable and accessible for LMICs; and (iii) acceptability of the vaccine by the general population. In addition to the above factors, rumors and misinformation about COVID-19 vaccination also contribute to the challenges. Therefore, we suggest that it is important for the government, healthcare workers, and the media to work in coordination and take the above factors into consideration to educate the individuals and increase the vaccination rate. As mentioned above, vaccination is the single most effective treatment available as of now that has shown to decrease the risk of hospitalization, severe illness, and death from COVID-19.
